# Activation of MEK1/2‐ERK1/2 signaling during NNK‐induced lung carcinogenesis in female A/J mice

**DOI:** 10.1002/cam4.652

**Published:** 2016-02-10

**Authors:** Keiko Yamakawa, Masanao Yokohira, Yuko Nakano, Sosuke Kishi, Shohei Kanie, Katsumi Imaida

**Affiliations:** ^1^ Departments of Pathology and Host‐DefenseOnco‐PathologyFaculty of MedicineKagawa UniversityKagawaJapan; ^2^ Toxicology LaboratoryTAIHO Pharmaceutical Co., Ltd.TokushimaJapan

**Keywords:** ERK1/2, *Kras* mutation, lung carcinogenesis, MEK1/2, nonsmall lung cancer

## Abstract

The extracellular signal‐regulated kinase 1/2 (ERK1/2) signaling pathway is activated by several growth factors and mitogens, and upregulation has been noted in many human cancers, including examples in the lung. In this study, to study the association of ERK1/2 activation with mutation of *Kras* encoding an upstream activator of ERK1/2 in lung premalignant lesions, we immunohistochemically examined expression of phosphorylated forms of ERK1/2 (pERK1/2) and MAP/ERK kinase 1/2 (pMEK1/2) proteins and correlation between ERK activation and mutation of *Kras* encoding an upstream activator of ERK1/2, in a mouse lung carcinogenesis model. Female 7‐week‐old A/J mice were administered a single dose of 4‐(methylnitrosamino)‐1‐(3‐pyridyl)‐1‐butanone (NNK), then maintained without additional treatment until sacrifice at week 52. Histopathologically, adenocarcinomas, adenomas and hyperplasias were observed in the lung. pMEK1/2 was expressed mostly in the cell cytoplasm in all three. In contrast, pERK1/2‐positive cells were also relatively rare in any histological types as compared with level of pMEK1/2 expression. However, pERK1/2‐positive cells in adenocarcinoma were still markedly more common than in hyperplasias and adenomas (~5‐fold, ~4‐fold; *P *< 0.01). Activating mutations of Kras gene at codons 12, 13 and 61 were detected in the majority of adenomas and adenocarcinomas, but without any significant relation to pERK1/2 expression. These results suggest that activation of ERK1/2 plays a key role in malignant transformation during lung carcinogenesis featuring *Kras* mutaion. Activation of ERK1/2 in lung premalignant lesions was little regardless of the mutation of *Kras*, and ERK1/2 activation in NNK‐induced mouse lung carcinogenesis may be regulated not only by *Kras* mutation but also other signaling pathway or regulatory factor.

## Introduction

Lung cancer is one of the most common causes of cancer mortality worldwide, and nonsmall cell lung cancers (NSCLCs) account for approximately 80% of the cases 1, 2. Recent studies have shown a high frequency of RAS mutations detected in many human cancers, including NSCLCs, where they are associated with unfavorable prognosis and poor sensitivity to epidermal growth factor receptor (EGFR) tyrosine kinase inhibitors 3, 4, 5. RAS is a molecule downstream of the EGFR signaling pathway, signals being transduced to the nucleus through phosphorylation of the extracellular signal‐regulated kinase 1/2 (ERK1/2) pathway (RAS‐Raf‐ MAP/ERK kinase 1/2 (MEK1/2) –ERK1/2 cascade), a mitogen‐activated protein kinase (MAPK) pathway playing a key role in the regulation of normal cell motility, proliferation, differentiation and survival 4, 6, 7, 8. Recent studies have suggested that activation of the ERK1/2 signaling pathway contributes to carcinogenesis, especially in promotion of malignant transformation, since: (1) mutationally activated *EGFR*,* RAS,* and *Raf* or overexpression of EGFR, which are upstream components of ERK1/2, have been detected at high frequency in human cancers including many in the lung 3, 4, 9, and (2) overexpression of the phosphorylated form of ERK1/2 (pERK1/2) appears frequent (>30%) in various human cancers and appears to have potential as a prognostic factor or a predictive factor for sensitivity to therapy 10, 11, 12, 13, 14, 15. MEK1/2 is phosphorylated by MEK kinases (MEKKs) including Raf, and the phosphorylated form of MEK1/2 (pMEK1/2) in turn phosphorylates tyrosine and serine/threonine residues in ERK1/2 3, 8. Thus, activation of ERK1/2 in human cancer cells is associated with activation of MEK 15. In a study using MEK1/2‐deficient mice, absence of MEK1/2 expression was found to be associated with a decreased incidence of lung tumors and increase in the survival rate 16. Thus, activations of RAS, MEK1/2, and ERK1/2 plays an important role in cancer malignancy through MAPK signaling pathway.

Rodent models of lung carcinogenesis are excellent tools for the study of the mechanisms underlying human NSCLC development, since the morphologies, histogenesis, and molecular characteristics of the induced primary lesions are similar to those humans 17. In particular, the female A/J mouse is sensitive to lung carcinogens, yielding a high incidence of with 4‐(methylnitrosamino)‐1‐(3‐pyridyl)‐1‐butanone (NNK), a tobacco‐specific nitrosamine component of tobacco smoke. *Kras* mutations, equivalent to examples found in human NSCLCs in tobacco smoking patients, have been detected at a high frequency in NNK‐induced lung tumors 18, 19, 20, 21, 22. Western blotting analysis showed levels of pERK1/2 protein in mouse lung tumors to be higher than in control lung tissue, again similar to human NSCLCs 23, 24. However, there are few the report about activations of ERK1/2 and MEK1/2 in lung proliferative or precancerous lesions.

In this study, to clarify the role of activations of MEK1/2 and ERK1/2 in lung tumorigenesis as well as malignant progression, we examined active mutations in exon 2 and 3 of the *Kras* gene and immunohistochemical expression of pERK 1/2 and pMEK1/2, downstream targets of Kras, using a mouse lung carcinogenesis model. Furthermore, in order to determine whether the extent of activation of pERK1/2 is influenced by Kras activation, we examined inter‐relationships during mouse lung carcinogenesis.

## Materials and Methods

### Animal treatment and tissue samples

For analysis of *Kras* mutations and immunohistochemistry, formalin fixed paraffin embedded lung neoplastic lesions (hyperplasia, adenoma, and adenocarcinoma) from 15 female A/J mice, administered a single dose of NNK (2 mg/0.1 mL saline/mouse, i.p.) at 7‐weeks of age and then maintained without additional treatment until sacrifice at week 52 25. The protocol of the experiment was approved by the Animal Care and Use Committee of Kagawa University and the animals were maintained in the Kagawa University Animal Facility according to the institutional animal care guidelines. They were housed in polycarbonate cages with white wood chips for bedding and given free access to drinking water and a basal diet (Oriental MF, Oriental Yeast Co., Ltd, Tokyo, Japan), under controlled conditions of humidity (60 ± 10%), lighting (12‐h light/dark cycle) and temperature (24 ± 2°C).

### Immunohistochemistry

Single‐ and double‐immunohistochemical staining was performed with the labeled streptavidin–biotin (LSAB) method, all staining processes from deparaffinization to counterstaining with hematoxylin being accomplished using an automated immunohistochemical stainer (Ventana HX Discovery system; Ventana Medical Systems, Tucson, AZ). For antigen retrieval, lung tissue sections were heated in RicoCC Buffer (Ventana Medical Systems) at 95°C for 30 min. Thereafter, the sections were incubated with the following primary antibodies: pERK1/2 (Thr202/Tyr204, rabbit monoclonal, 1:200 dilution for 1 h at 42°C, Cell Signaling Technology, MA, USA), pMEK1/2 (Ser221, rabbit monoclonal, 1:50 dilution for 12 h at room temperature, Cell Signaling Technology) and proliferating‐cell nuclear antigen (PCNA, mouse monoclonal, 1:200 dilution for 12 h at room temperature, Dako, Glostrup, Denmark,). Incubation with PCNA primary antibody was performed using a MoMap Kit (Ventana Medical Systems), without secondary antibody. Incubation with biotinylated goat anti‐rabbit IgG secondary antibody (Vector Laboratories, Inc., CA) for pMEK1/2 and pERK1/2 was performed for 30 min.

For single immunohistochemical staining, pMEK1/2 and pERK1/2 primary antibody binding sites were stained brown using a diaminobenzidine (DAB)–HRPO hydrogen peroxidase (H_2_O_2_) solution (DAB Map Kit, Ventana Medical Systems). For double‐immunohistochemical staining of pMEK1/2 and pERK1/2, pMEK1/2 primary antibody binding sites were stained brown using a DAB Map Kit and pERK1/2 primary antibody binding was indicated with a fast red chromogen that reacts with alkaline phosphatase (Red Map Kit, Ventana Medical Systems). For double‐immunohistochemical staining with pERK1/2 and PCNA, pERK1/2 primary antibody binding sites were stained brown using DAB Map Kit and PCNA primary antibody binding sites were stained red using Red Map Kit.

### Immunohistochemistry scoring

#### Single‐immunohistochemistry for pMEK1/2 and pERK1/2

Expression levels of pERK1/2 and pMEK1/2 were assessed semiquantitatively with a scoring system taking into account both intensity of immunostaining within tumor cell cytoplasm (pMEK1/2) or the cytoplasm/nucleus (pERK1/2) and the number of positively stained tumor cells. Intensity was classified with a scale of 0–3 (0, negative; 1, weak positive; 2, moderate positive; 3, strong positive). Negative was defined as a level similar to that in normal epithelial cells. Moderately or strongly positive was defined as a level identifiable in low‐power fields (4 × objective lens) and weakly positive as requiring high‐power observation (20 × objective lens). The number of positive stained cells was scored from 0 to 4 (0, no stained cells; 1, <10% positive cells; 2, 10–50% positive cells; 3, 50–80% positive cells; 4, >80% positive cells). The final positive score was obtained by multiplying the score for the number of positively stained tumor cells by the intensity (range 0–12).

#### Double‐immunohistochemistry for pMEK1/2 and PCNA with pERK1/2

Each expression level was assessed semiquantitatively. Staining intensity for pMEK1/2 and pERK1/2 was classified with a scale of 0–3 (negative: 0, weak positive: 1, moderately positive: 2, strong positive: 3), similar to single‐immunohistochemical staining. The number of PCNA positive stained cells were classified with a scale of 0–3 (no positive cell: 0, weak positive: 1, moderately positive: 2, strong positive: 3). Weak was defined as only <10% positive cells, moderately as10–50% positive cells and strongly as >50% positive cells. Positive for immunohistochemistry was concluded with an expression level of 2 or 3, and negative with an expression level of 0 or 1.

### Analysis of Kras mutation

For mutation analysis of *Kras* codon 12, 13, and 61, lung tumor DNA extraction and condition of amplification by polymerase chain reaction (PCR) were performed as previously described 22. PCR primers were as described by Kitahashi et al. 21. Briefly, lung tumor DNA was extracted from one unstained paraffin section using laser capture microdissection, and subsequently amplified by two‐step PCR. The second PCR products were purified with SUPREC‐02 (Takara Bio Inc., Shiga, Japan) or QIAquick PCR Purification Kit (Qiagen GmbH, Hilden, Germany). Subsequently, 2–3 ng purified products were directly sequenced using the BigDye^®^ Terminator v1.1 Cycle Sequencing kit (Applied Biosystems, Foster City, CA) with second PCR primers, BigDye^®^ XTerminator Purification Kit (Applied Biosystems) and an ABI PRISM^®^ 310 Genetic Analyzer (Applied Biosystems).

### Statistical Analysis

Single‐immunohistochemical positive scores were compared using the Kruskal–Wallis test and Steel–Dwass test. The correlation between *Kras* mutation and pERK1/2 expression was evaluated with the nonparametric Mann–Whitney *U*‐test and immunohistochemical staining intensity of pERK1/2 and pMEK1/2, and pERK1/2 and PCNA with the Spearman rank correlation test. The association of histological type with double‐immunohistochemical staining intensity was evaluated with the chi‐squared for independence test. Differences were considered statistically significant at *P *< 0.05.

## Results

### *Expression of pMEK1/2 in lung neoplastic lesions*

**Figure 1:**
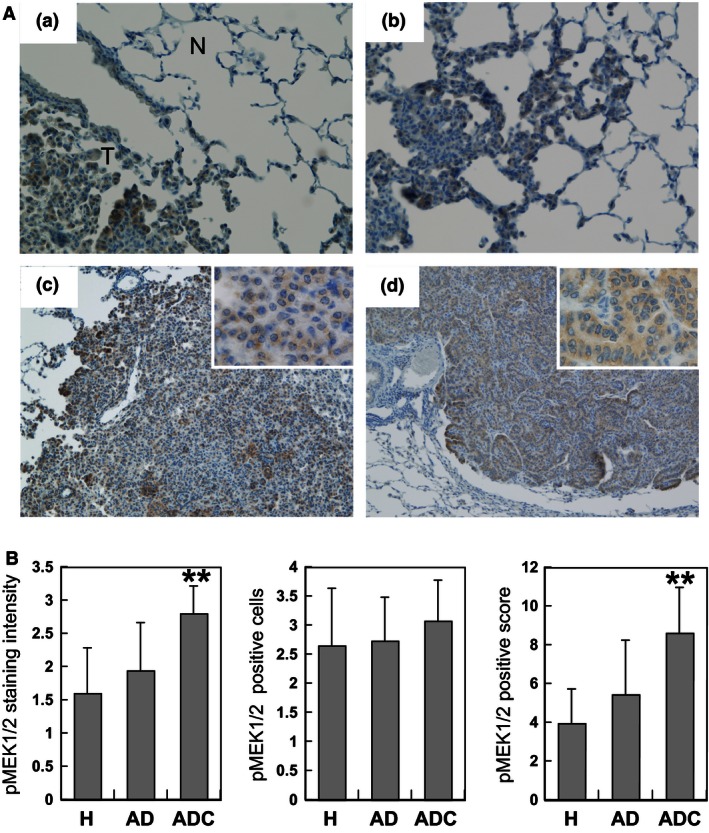
Immunohistochemical expression of phosphorylated MEK1/2 (pMEK1/2) in 4‐(methylnitrosamino)‐1‐(3‐pyridyl)‐1‐butanone ‐induced mouse lung tumors. (A) Immunohistochemical staining pattern of pMEK1/2 with tumor progression: (a) surrounding normal tissue (N) of a tumor (T); (b) hyperplasia negative or weakly positive with pMEK1/2; (C) adenoma; (D) adenocarcinoma moderate or strongly positive for pMEK1/2. Magnification ×200 (a, b); ×100 (c, d); ×400 inset (c, d). (B) Scores of staining intensity, numbers of positive cells and their multiplication (positive score) for pMEK1/2 in hyperplasia (H), adenoma (AD) and adenocarcinoma (ADC). Shown are averages and standard deviations (error bars). **P < 0.01 indicates a significant difference from H and AD.

We analyzed immunohistochemical expression of pMEK1/2 in 20 hyperplasias, 49 adenomas, and 15 adenocarcinomas (see Fig. 1A). Surrounding tissues of tumor lesions showed almost no or only weak expression of pMEK1/2. The level was already increased mostly in tumor cell cytoplasm with hyperplasias and adenomas, but the highest pMEK1/2 positive scores were found in adenocarcinomas (8.6 vs. 4.0 and 5.4, respectively; *P* < 0.01, Fig. 1B), due to strong staining intensity rather than the number of pMEK1/2‐positive cells.

### Expression of pERK1/2 in lung neoplastic lesions

**Figure 2:**
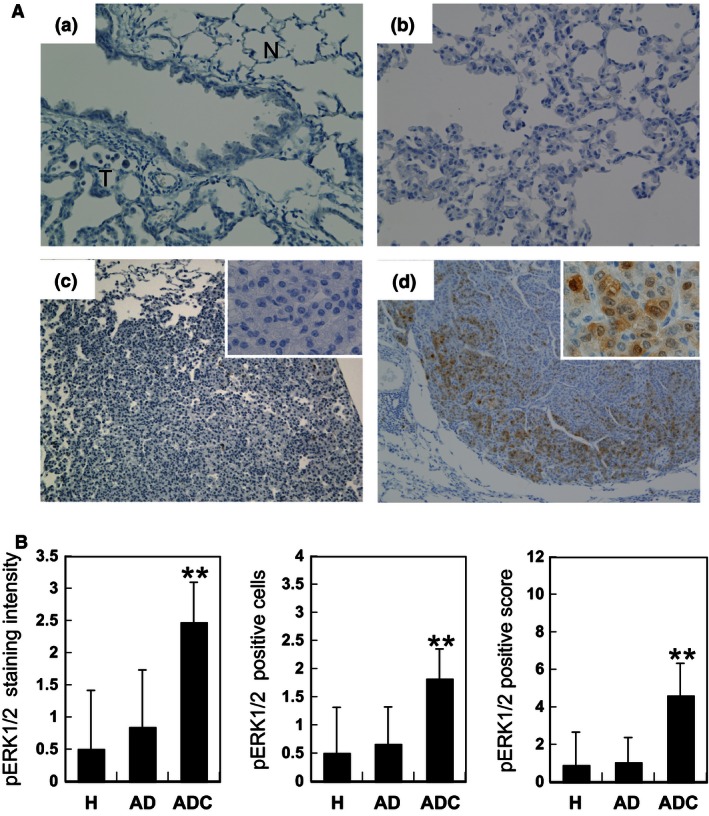
Immunohistochemical expression of phosphorylated extracellular signal‐regulated kinase 1/2 ( ERK1/2) (pERK1/2) in 4‐(methylnitrosamino)‐1‐(3‐pyridyl)‐1‐butanone ‐induced mouse lung tumors. (A) Immunohistochemical staining pattern for pERK1/2 with tumor progression: (a) surrounding normal tissue (N) of a tumor (T); (b) hyperplasia; (c) adenoma, negative for pERK1/2; (d) adenocarcinoma intensely stained for pERK1/2, in both nuclei and cytoplasm. Magnification ×200 (a, b); ×100 (c, d); ×400 inset (c, d). (B) Scores of staining intensity, number of positive cells and their multiplication (positive score) for pERK1/2 in hyperplasia (H), adenoma (AD) and adenocarcinoma (ADC). Shown are averages and standard deviations (error bars). **P < 0.01 indicates a significant difference from H and AD.

**Figure 3:**
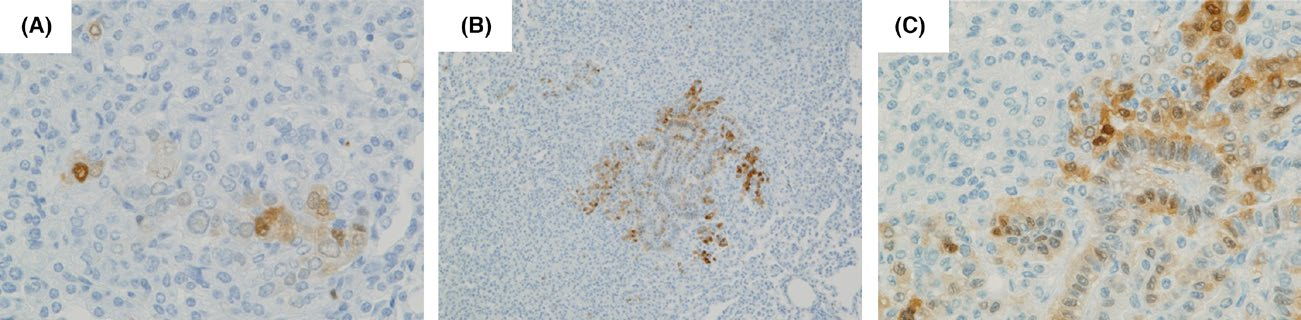
Immunohistochemistry findings for phosphorylated extracellular signal‐regulated kinase ½ (ERK1/2) (pERK1/2) in 4‐(methylnitrosamino)‐1‐(3‐pyridyl)‐1‐butanone ‐induced mouse adenomas. (A) A few cells with swelling and irregularly shaped nuclei are stained for pERK1/2. (B, C) Note higher level of pERK1/2 expression associated with a papillary architecture. Magnification: ×100 (B) and ×400 (A, C).

We analyzed immunohistochemical expression of pERK1/2 in 26 hyperplasias, 50 adenomas, and 17 adenocarcinomas (see Fig. 2A). Surrounding normal tissues of tumors, hyperplasias, and adenomas showed almost no or partial expression of pERK1/2. In adenocarcinomas, both the nuclei and cytoplasm were intensely stained, especially at the periphery. The pERK1/2 positive score for adenocarcinomas was remarkably high as compared to those for hyperplasias and adenomas (4.6 vs. 0.9 and 1.0; *P* < 0.01, Fig. 2B). Similarly, both pERK1/2‐staining intensity and positive cells in adenocarcinomas were remarkably elevated as compared to values for hyperplasias and adenomas (Fig. 2B). In adenomas, tumor cells with characteristic of malignancy, like an atypical and swollen nucleus, expressed pERK1/2 more strongly than surrounding adenoma cells (Fig. 3A). Furthermore, tumor cells forming papillary architecture tended to be positively stained (Fig. 3B and C).

### Inter‐relationship of expression of pMEK1/2 and pERK1/2 in lung neoplastic lesions

**Figure 4:**
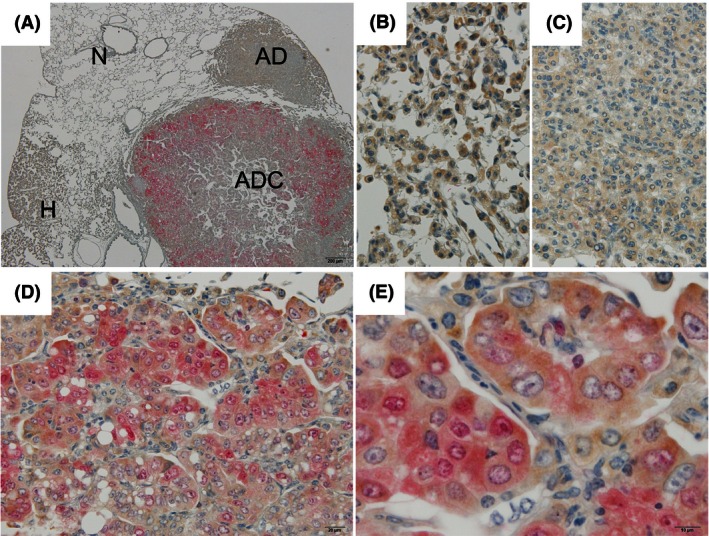
Double‐immunohistochemistry findings for phosphorylated MEK1/2 (pMEK1/2) and phosphorylated extracellular signal‐regulated kinase 1/2 (ERK1/2) (pERK1/2) in 4‐(methylnitrosamino)‐1‐(3‐pyridyl)‐1‐butanone ‐induced lung proliferative lesions. (A) Low magnification of normal (N), hyperplasia (H), adenoma (AD), and adenocarcinoma (ADC). High magnification of (B) hyperplasia; (C) adenoma, positive for only pMEK1/2 (brown); (D, E) adenocarcinoma, positive for both of pMEK1/2 and pERK1/2 (red). Magnification: ×40 (A), ×400 (B–D) and ×1000 (E).

Double‐immunohistochemical staining for pMEK1/2 and pERK1/2 was performed on a total of 64 lung tumors (15 hyperplasias, 36 adenomas, and 13 adenocarcinomas) (see Fig. 4). In the hyperplasias and adenomas, although the level of pMEK1/2 expression in a large number of tumor cells was higher than in normal cells, pERK1/2 expression was scant. In adenocarcinomas, the expression of pERK1/2 was observed in a fraction of pMEK1/2 positive stained cells. In accordance with a high level of pMEK1/2 expression in the cytoplasm of adenocarcinoma cells with an increased nuclear/cytoplasm ratio and irregularly shaped nuclei and prominent nucleoli, in these adenocarcinoma cells cytoplasm and nucleus were intensely stained for pERK1/2. The staining intensity of pERK1/2 increased in parallel with that of pMEK1/2 (Spearman correlation coefficient: 0.59, *P* < 0.0001, Table 1). A comparison of the expression of both, the pMEK1/2‐pERK1/2 expression pattern was significantly associated with histological type (*P* < 0.0001, Table 2). In contrast to adenocarcinomas being positive for both pMEK1/2 and pERK1/2, 40% of hyperplasias and 88% of adenomas were pMEK1/2 positive ‐pERK1/2 negative. Thus, in hyperplasias and adenomas, marked differences in levels of pMEK1/2 and pERK1‐2 expressions were observed.

**Table 1:** Correlation between levels of expressions of pMEK1/2 and pERK1/2 in 4‐(methylnitrosamino)‐1‐(3‐pyridyl)‐1‐butanone (NNK)‐induced mice lung tumors using double immunohistochemistry

pERK1/2 expression	pMEK1/2 expression			
	0	1	2	3
0	0	9	23	3
1	0	1	2	8
2	0	1	3	3
3	0	0	4	7

*P* < 0.0001, Spearman rank correlation test.

Spearman correlation coefficient: 0.59.

**Table 2:** Association between pMEK1/2 and pERK1/2 expressions and histological type of 4‐(methylnitrosamino)‐1‐(3‐pyridyl)‐1‐butanone (NNK)‐induced mouse lung tumors using double immunohistochemistry

Histological type	*n*	pMEK1/2 expression	a	a	b	b
		pERK1/2 expression	a	b	a	b
Hyperplasia	15		7 (47%)	1 (7%)	6 (40%)	1 (7%)
Adenoma	36		3 (9%)	0 (0%)	29 (88%)	4 (12%)
Adenocarcinoma	13		0 (0%)	0 (0%)	1 (8%)	12 (92%)

*P* < 0.0001, Chi‐square for independence test, Histological type vs. pMEK1/2 and pERK1/2 expression.

a negative with staining intensity scores of 0 and 1;

b positive with scores of 2 and 3.

### Inter‐relationship of expression of pERK1/2 and PCNA in lung neoplastic lesions

**Figure 5:**
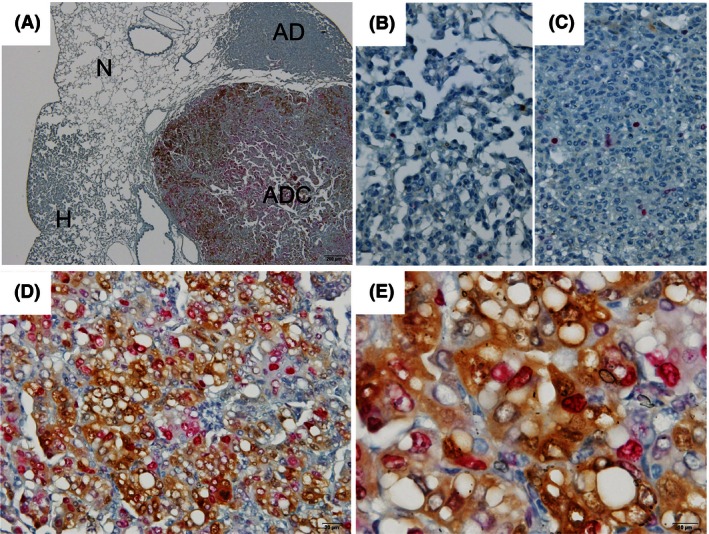
Double‐immunohistochemistry findings for phosphorylated extracellular signal‐regulated kinase 1/2 (ERK1/2) (pERK1/2) and proliferating‐cell nuclear antigen (PCNA) in 4‐(methylnitrosamino)‐1‐(3‐pyridyl)‐1‐butanone ‐induced lung proliferative lesions. (A) Low magnification of normal (N), hyperplasia (H), adenoma (AD), and adenocarcinoma (ADC). High magnification of (B) hyperplasia; (C) adenoma, negative for pERK1/2 (brown) and PCNA (red); (D, E) adenocarcinoma, positive for both. Magnification: ×40 (A), ×400 (B–D) and ×1000 (E).

In order to examine the relationship between cell proliferation and ERK1/2 activation in mouse lung tumors, double‐immunohistochemical staining for pERK1/2 and PCNA was performed in a total of 61 lesions (13 hyperplasias, 34 adenomas, and 14 adenocarcinomas) (see Fig. 5). In normal, hyperplasia and adenoma, the expression of both pERK1/2 and PCNA was negative or observed locally. In contrast, PCNA positive tumor cells were remarkably increased in pERK1/2 strongly positive adenocarcinomas. However, in individual pERK1/2‐positive adenocarcinoma cells, the expression of PCNA was not always high, and various adenocarcinoma cells with different expression (e.g., only PCNA or only pERK1/2 or either strong and weak) were also admixed (Fig. 5E). The expression of PCNA was positive correlated with expression of pERK1/2 (Spearman correlation coefficient: 0.63, *P* < 0.0001, Table 3). On comparison across lesions, 70% or more of hyperplasias and adenomas were negative for both. In contrast, both were strongly expressed in numerous adenocarcinomas and the pERK1/2‐PCNA expression pattern was significantly associated with histological type (*P* < 0.0001, Table 4).

**Table 3:** Correlation between pERK1/2 and PCNA expressions in 4‐(methylnitrosamino)‐1‐(3‐pyridyl)‐1‐butanone (NNK)‐induced mouse lung tumors using double immunohistochemistry

pERK1/2 expression	PCNA expression			
	0	1	2	3
0	0	25	2	0
1	0	9	4	1
2	0	5	5	2
3	0	1	1	6

*P* < 0.0001, Spearman rank correlation test.

Spearman correlation coefficient: 0.63.

**Table 4:** Association between pERK1/2 and PCNA expression and histological type of 4‐(methylnitrosamino)‐1‐(3‐pyridyl)‐1‐butanone (NNK)‐induced mouse lung tumors using double immunohistochemistry

Histological type	*n*	pERK1/2 expression	a	a	b	b
		PCNA expression	a	b	a	b
Hyperplasia	13		10 (77%)	0 (0%)	3 (23%)	0 (0%)
Adenoma	34		24 (71%)	6 (18%)	1 (3%)	3 (9%)
Adenocarcinoma	14		0 (0%)	1 (7%)	2 (14%)	11 (79%)

*P* < 0.0001, Chi‐square for independence test, Histological type vs. pERK1/2 and PCNA expression.

a Negative with staining intensity scores of 0 and 1.

b Positive with scores of 2 and 3.

### Kras gene alteration in lung lesions and correlation with pERK1/2 expression

Mutation analysis of the *Kras* gene was performed on a total of 25 adenomas and 13 adenocarcinomas. Activating mutations of *Kras* gene at any one of codon 12, 13, and 61 were detected in 19 adenomas (76%), and 10 adenocarcinomas (77%), no significant variation being evident with the histological type. The *Kras* mutations at codon 12 were frequently detected in both of adenoma (76%) and adenocarcinoma (61%), while *Kras* mutations at codon 13 and 61 were infrequent (Table 5). Mutations were mostly G/C→A/T transitions, and G12D mutation was most frequent type of *Kras* mutation (Table 6).

**Table 5:** Incidences of mutations with amino acid substitution of the *Kras* gene codon 12, 13, and 61

Histological type	*n*	Codon 12	Codon 13	Codon 61
Adenoma	25	19 (76%)	0 (0%)	1 (4%)
Adenocarcinoma	13	8 (61%)	2 (15%)	1 (8%)

**Table 6:** Mutation patterns for the *Kras* codon 12, 13, and 61

Histological type	Codon	Nucleotide change	Amino acid change	Frequency
Adenoma	12	GGT→GAT	G12D	18/19
		GGT→TGT	G12C	1/19
	61	CAA→TAA	Q61X	1/1
Adenocarcinoma	12	GGT→GAT	G12D	6/8
		GGT→AAT	G12N	1/8
		GGT→GTT	G12V	1/8
	13	GGC→GAC	G13D	2/2
	61	CAA→CGA	Q61R	1/1

To investigate whether the level of pERK1/2 expression is influenced by *Kras* mutation status, the 24 adenomas and 13 adenocarcinomas were evaluated for grading of positive scores of single‐immunohistochemical staining for pERK1/2 (classified into four categories, negative (positive score 0), weak (positive score 1 and 2), moderate (positive score 3 and 4), and strong (positive score 6≤)) and *Kras* mutation status. The vast majority of adenomas harboring *Kras* mutation, were negative or had only faint expression of pERK1/2. Furthermore, in majority of adenocarcinomas, the expression of pERK1/2 was observed moderately or strongly, regardless of where they harbor active mutation of *Kras* gene at codon 12, 13 and 61. The level of pERK1/2 expression did not correlate with the *Kras* mutation status in either adenomas or adenocarcinomas (Table 7).

**Table 7:** Correlation between *Kras* mutation and pERK1/2 expression in mouse lung adenomas and adenocarcinomas

Histological type	*n*	*Kras* mutation status			pERK1/2 expression				
		Codon 12, 13, and 61		*P*	Negative	Weak	Moderate	Strong	*P*
Adenoma	24	Mutation	18 (75%)		8	9	1	0	
		Wild	6 (25%)		3	2	1	0	0.51^b^
Adenocarcinoma	13	Mutation	10 (77%)		0	2	2	6	
		Wild	3 (23%)	0.61^a^	0	0	2	1	0.35^c^

a Fisher's exact probability test, Histological type vs. *Kras* mutation.

b Mann‐Whitney's *U* test, *Kras*. mutation versus pERK1/2 expression in adenoma.

c Mann‐Whitney's *U* test, *Kras* mutation vs. pERK1/2expression in adenocacinoma.

## Discussion

In the present, analysis using immunohistochemistry techniques, activation of the MAPK pathway appeared frequent in NNK‐induced mouse lung tumors. There have been numerous reports of MAPK activation found by western blotting, especially involving ERK1/2 phosphorylation, in human and animal lung tumors. In human lung cancer, levels of pERK1/2 in adenocarcinomas are reported to be significantly higher than in squamous cell carcinomas 10. In an earlier NNK‐induced mouse lung carcinogenesis study, the expression levels of pERK and PCNA, assessed as a marker of cell proliferation, were higher in adenocarcinomas than in adenomas by immunohistochemical staining, suggesting a positive correlation between ERK activity and cell division 26. Immunohistochemical staining is useful for detection of target protein expression, localization and relationship with histopathology.

In our NNK‐induced mouse adenocarcinomas, both intensity of immunostaining of pERK1/2 within tumor cells and the number of positively stained cells were increased markedly as compared to hyperplasias and adenomas. Similarly, in adenocarcinomas with strong pERK1/2, the level of PCNA expression was increased as compared to that in hyperplasias and adenomas without activated ERK1/2. Sustained ERK signaling may promote cell proliferation as a consequence of phosphorylation or stabilization of genes involved in cell cycle entry or repression of inhibitors of proliferation 6, 9, 27, 28. In the NNK‐induced mouse lung carcinogenesis model, the correlation between increase in cell proliferation and activation of ERK1/2 suggests that ERK1/2 activation is involved in the process of malignant progression (adenoma to adenocarcinoma). Furthermore, in mixed solid‐papillary adenomas, ERK activation appeared associated with papillary growth of cuboidal to columnar cells. Because tumors partly featuring this morphology seem to be more aggressive, 26 the cells with activated ERK1/2 and papillary growth may be transformed to a higher level of malignancy. ERK activation in adenomas may therefore be a reflection of the early stage of the process of malignant progression. Thus, these results raise the possibility that inhibition of ERK activation in lung precancerous lesions may prevent malignant progression (adenoma to adenocarcinoma). A number of studies have reported that in vitro or in vivo pharmacologic inhibition of ERK1/2 using a selective MEK1/2 inhibitor can cause decrease in pERK1/2 and cell proliferation, and treatment of MEK inhibitor lead lung tumor regression and a decrease in pERK1/2 in *KRAS* mutant mouse lung cancer model 24, 29. MEK inhibitor may be effective for prevention of malignant transformation in premalignant lesion through RAS‐MEK1/2‐ ERK1/2 signaling pathway as well as lung adenocarcinoma chemoprevention.

In this study, there was no significant correlation between *Kras* mutation and level of pERK1/2 expression. *RAS* mutation is an important cause of activation of ERK signaling in a variety of human cancers 3, 4, 9. In human lung cancer, correlation between *KRAS* mutation and pERK 1/2 expression is reported to be strong 14. However, no significant correlations has been noted between mutation of upstream components of ERK (e.g., EGFR, RAS and RAF) and pERK1/2 expression in human and mouse cancers 11, 13, 21. In rodent NNK‐induced lung tumors, a high frequency of *Kras* mutations is already detected in hyperplasias, and a correlation with proliferative activity appears lacking 30. In this study, the level of ERK1/2 activation in the vast majority of adenoma was very low, although these adenomas demonstrated a high frequency of *Kras* mutation and upregulation of activated MEK1/2. The results suggest that, in the mouse lung carcinogenesis model with chemical carcinogens, *Kras* mutation is a very early event during lung tumorigenesis, but ERK activation may not be necessarily caused by activation of upstream components of ERK1/2 (e.g., KRAS and MEK1/2) at such an early stage. In human *KRAS* mutant lung adenocarcinoma, downstream effectors of KRAS interacted with Akt/mTOR signaling pathway, receptors of tyrosine kinase and adaptor proteins, and the activation levels of ERK1/2 in two‐thirds of lung adenocarcinomas with *KRAS* mutation were comparable with that of wild‐type 31. Similarly, The activation of ERK1/2 in NNK‐induced mouse lung adenocarcinomas may be caused not only by *Kras* mutation but also crosstalk with activations of various signaling pathways and effectors.

Nevertheless, the reason why the level of pERK1/2 expression was very low in adenomas harboring *Kras* mutation remains unclear. We think that it is also necessary to consider the expression level of activated Ras protein in adenoma as a factor that affects the activation of ERK1/2, not only the presence of a mutation in the Ras gene. Furthermore, ERK signaling is controlled by complex regulatory mechanisms that depend on activation or inactivation of core components of the pathway, ERK scaffolding proteins and signaling modulators 6, 8, 32. Mizumoto et al. have suggested that dysregulation of complex regulatory mechanisms are involved in ERK activation in endometrial cancers, where there is similarly a lack of ERK activation in the presence of *KRAS* mutations 13. Several negative regulators of ERK signaling have been identified, including Sprouty, SPRED, protein phosphatase 2A (PP2A), protein tyrosine phosphatase (PTP), and dual specificity phosphatase (DUSP) which are inhibitors of RAS or RAF activation and protein phosphatases in the ERK pathway 7, 8, 9, 32, 33. We previously analyzed gene expression in NNK‐induced mouse lung adenocarcinomas at 78 weeks, using an oligonucleotide microarray. Expression of *Ptp* and *Dusp* families dephosphorylating MAPKs including ERK1/2 were found to be decreased (−2.1 to −4.9‐fold) in lung adenocarcinomas when compared with normal adjacent tissue (data not shown). Since these phosphatases are negative regulators of ERK1/2 activation, such downregulation of negative regulators of ERK1/2 may be one of factors in the activation of ERK1/2 in adenocarcinomas, although examples induced by NNK at 52 weeks have not been analyzed so far. The multiple negative regulator feedback loops in the ERK pathway appear to play key roles in activation of ERK in human cancer. Furthermore, it appears that imbalance of activating signals and negative regulation mechanisms in ERK signaling can give rise to a more aggressive phenotype of tumor cells 9, 13, 34, 35. It is not clear whether other negative regulators are involved in the inactivation of ERK1/2 in adenomas harboring *Kras* mutations in this mouse model, but the absence of pERK1/2 in NNK‐induced adenomas suggests that regulatory mechanisms of ERK1/2 phosphorylation may function in lung precancerous lesions.

In conclusion, the present results indicate that activation of ERK1/2 plays a key role in malignant transformation during mouse NNK‐induced lung carcinogenesis, apparently independent of *Kras* mutation. In particular, ERK1/2 activation in this mouse lung premalignant lesion was not necessarily caused by activation of upstream components of ERK1/2. In NNK‐induced mouse lung carcinogenesis, there is a time lag between Kras and ERK1/2 activation. Thus (1) ERK1/2 activation may be regulated by a number of factors other than *Kras* mutation, and (2) activation of ERK1/2 in mouse lung premalignant lesions is controlled by negative regulators. Further elucidation of the mechanisms of ERK1/2 activation and assessment of the balance between activating signals and negative regulation of ERK signaling are warranted.

## Conflict of Interest

K. Imaida received research found from TAIHO Pharmaceutical Co., Ltd. Other authors have no conflict of interest. S. Kanie is employee of TAIHO Pharmaceutical Co., Ltd.
